# Nucleic acid purification from plants, animals and microbes in under 30 seconds

**DOI:** 10.1371/journal.pbio.2003916

**Published:** 2017-11-21

**Authors:** Yiping Zou, Michael Glenn Mason, Yuling Wang, Eugene Wee, Conny Turni, Patrick J. Blackall, Matt Trau, Jose Ramon Botella

**Affiliations:** 1 Plant Genetic Engineering Laboratory, School of Agriculture and Food Sciences, The University of Queensland, St.Lucia, Australia; 2 Centre for Personalized Nanomedicine, Australian Institute for Bioengineering and Nanotechnology, The University of Queensland, St.Lucia, Australia; 3 Queensland Alliance for Agriculture and Food Innovation, The University of Queensland, St.Lucia, Australia; National Cancer Institute, United States of America

## Abstract

Nucleic acid amplification is a powerful molecular biology tool, although its use outside the modern laboratory environment is limited due to the relatively cumbersome methods required to extract nucleic acids from biological samples. To address this issue, we investigated a variety of materials for their suitability for nucleic acid capture and purification. We report here that untreated cellulose-based paper can rapidly capture nucleic acids within seconds and retain them during a single washing step, while contaminants present in complex biological samples are quickly removed. Building on this knowledge, we have successfully created an equipment-free nucleic acid extraction dipstick methodology that can obtain amplification-ready DNA and RNA from plants, animals, and microbes from difficult biological samples such as blood and leaves from adult trees in less than 30 seconds. The simplicity and speed of this method as well as the low cost and availability of suitable materials (e.g., common paper towelling), means that nucleic acid extraction is now more accessible and affordable for researchers and the broader community. Furthermore, when combined with recent advancements in isothermal amplification and naked eye DNA visualization techniques, the dipstick extraction technology makes performing molecular diagnostic assays achievable in limited resource settings including university and high school classrooms, field-based environments, and developing countries.

## Author summary

Nucleic acid amplification has proven to be indispensable in laboratories around the world for a myriad of applications from diagnostics to genotyping. The first step in any application aiming to amplify DNA or RNA is the extraction of nucleic acids from a complex biological sample; a task traditionally requiring specialised equipment, trained technicians, and multiple liquid handling steps. It is this complexity of current nucleic acid isolation methods that limit the use of many DNA amplification technologies outside of the modern laboratory environment. Therefore, in this study, we investigated new materials and approaches to simplify nucleic acid extraction. We found that cellulose-based filter paper can be used to rapidly bind nucleic acids, retain them during a short washing step to remove contaminants, and then elute them directly into the amplification reaction. We then adapted the cellulose filter to create a dipstick that can be used to purify nucleic acids from a wide range of plant, animal, and microbe samples in less than 30 seconds without the need for any specialised equipment. The speed and simplicity of our method makes it ideally suited for nucleic acid amplification-based applications both within and outside the laboratory, including limited resource settings such as remote field sites, developing countries, and teaching institutions.

## Introduction

The ability to amplify and detect specific DNA sequences is a powerful tool routinely used for a wide variety of applications including disease diagnostics, qualitative trait loci (QTL) selection and mutant screening. In diagnostic applications, nucleic acid-based analysis has many advantages over more traditional methods such as enzyme or antibody-based assays offering increased sensitivity, faster sample-to-answer results, and flexibility as it can be rapidly modified to meet new challenges as they arise [1]. However, the major bottleneck preventing the widespread adoption of molecular diagnostics outside the modern laboratory environment is the requirement to purify nucleic acids from samples, which is a complex task that traditionally requires trained technicians and involves many liquid handling steps [2–4].

The demand for simpler and more rapid nucleic acid purification methods resulted in the expansion of commercially available solid-phase extraction kits. A large majority of these kits are based on the binding of nucleic acids to a solid silica support in the presence of a chaotropic salt [5–8]. Contaminants are then removed by a series of wash and centrifugation steps before finally eluting the nucleic acids from the silica in a low salt solution. Commercially available paramagnetic beads with a variety of different functionalised surface chemistries designed to capture and purify nucleic acids have become available, removing the need for centrifugation [9–11]. In these systems, a magnet is used to attract and hold the paramagnetic beads to the side of the tube to allow supernatant removal during the wash and elution steps. Even though paramagnetic particle-based nucleic acid purification is relatively fast (approximately 10 minutes) and does not require electrical equipment, it is still too complicated for applications that are performed outside the modern laboratory environment such as field-based point-of-need (PON) assays.

Recent publications have reported rapid nucleic acid extraction using different types of membranes including aluminium oxide, the cellulose-based Flinders Technology Associates (FTA) cards (GE Healthcare, USA), and the silica-based Fusion 5 filters (GE Healthcare, USA) [12–18]. These new methods simplified the nucleic acid purification process by eliminating the need for a separate nucleic acid elution step by directly amplifying the nucleic acid off the membrane. This is an advantage over many of the other solid-phase extraction techniques as either the surface chemistries of the matrix or the residual reagents attached to them (e.g., ethanol, chaotropic salts) inhibit DNA amplification [17,19]. However, despite eliminating the elution step, all of these methods require relatively complex fabrication or experimental set ups, multiple pipetting steps, or electrical equipment to help purify the nucleic acids, which, again, limit their usefulness for field-based assays.

Cellulose-based DNA binding is ideal for molecular diagnostics as it is inexpensive, portable, disposable, and easily modified [20–22]. Therefore, we set out to develop a nucleic acid purification method using cellulose paper that does not require any complex fabrication or specialized equipment such as pipettes and centrifuges. Herein, we describe a simple, equipment-free method that can purify nucleic acids from a wide range of plant, animal, and microbe samples within less than 30 seconds, and is therefore equally suited to nucleic acid-based applications both within and outside the modern laboratory environment.

## Results

### Whatman No.1 filter paper can entrap and retain DNA after washing

**Fig 1:**
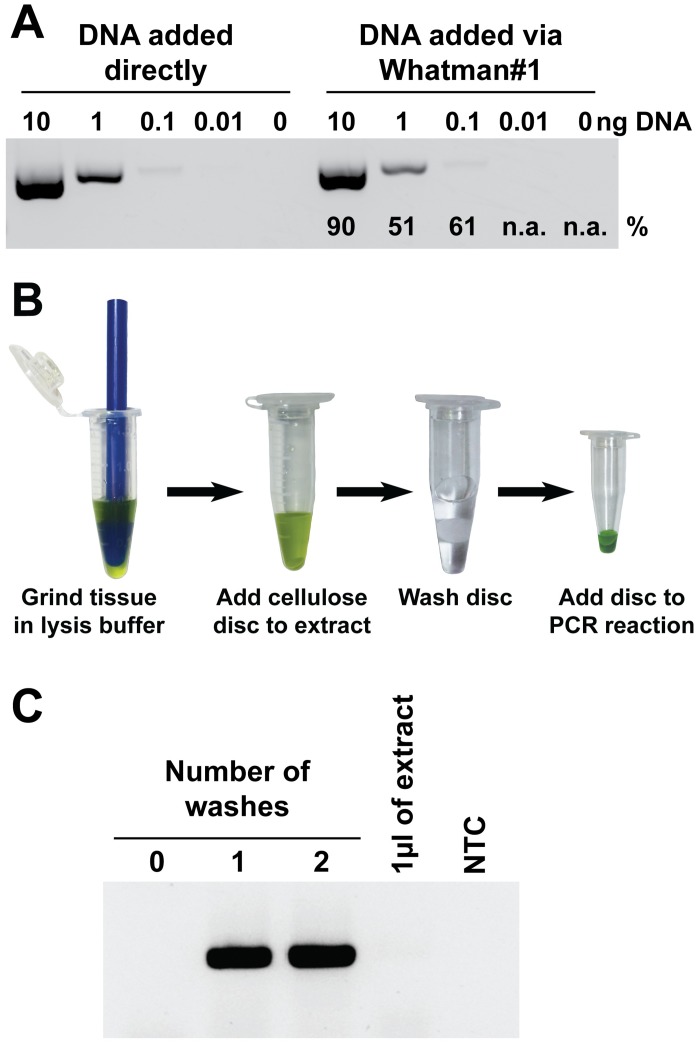
Cellulose-based paper can be used to capture and purify nucleic acids. (A) 1 μl of purified DNA at different concentrations (0, 0.01, 0.1, 1, or 10 ng/μl) was pipetted directly onto a Whatman No.1 disc (3 mm diameter) and then washed in 200 μl 10 mM Tris (pH 8) before adding the disc to a PCR reaction. As a control 1μl of the same DNA solutions were directly added to the PCR reactions. The band intensities achieved with cellulose discs relative to direct DNA addition appear below each band. (B) An outline of the nucleic acid purification method using Whatman No.1 discs. Tissue is ground in a 1.5 ml eppendorf tube with a plastic pestle in the presence of extraction buffer. Nucleic acids are captured by a 3-mm diameter Whatman No.1 disc. The disc is then transferred to a tube containing wash buffer for one minute to remove contaminants present in the crude extract before transferring it to the tube containing the amplification reaction. The PCR reaction is performed without removing the disc from the tube. (C) Whatman No.1 discs were immersed in an *Arabidopsis thaliana* leaf extract before being washed once, twice, or not at all prior to amplification. As controls, 1 μl of crude extract or water (NTC) was added directly to the PCR reaction. n.a., no amplification; NTC, no template control.

To develop a simple nucleic acid purification method that does not require modern laboratory facilities, we first investigated the ability of a number of cationic chemicals that could potentially help to capture anionic DNA and RNA by spotting them onto a piece of Whatman No.1 paper (GE Healthcare, USA). We found that a number of compounds, including chitosan and polyethylenimine (PEI), showed a strong ability to bind nucleic acids (S1A Fig) and were further tested for their ability to capture genomic DNA that could be directly amplified from the modified cellulose in a PCR reaction. In these experiments, none of the chemical treatments examined produced reproducible amplification. However, we noted that the control, unmodified Whatman No.1 paper, consistently resulted in strong amplification (S1B Fig). The ability of cellulose-based paper to entrap or adsorb DNA under specific conditions has been extensively reported, but its use has been limited to storage or transport and not for nucleic acid purification purposes under nonprecipitating conditions [10,11,23–25]. We further examined the efficiency at which Whatman No.1 can capture DNA and retain it during a brief (one minute) wash prior to DNA amplification directly from the paper. Our results show that the Whatman No.1 has a relatively high efficiency, as the amplification results were comparable to that observed when an identical amount of DNA template was added directly to the PCR reaction (Fig 1A). These results suggest that cellulose can efficiently bind, or at least entrap, DNA and does not inhibit the amplification reaction.

We then devised a simple nucleic acid purification method (outlined in Fig 1B and described in detail in the Material and methods section) to test whether it was possible to remove PCR-inhibiting chemical/biological contaminants present in a plant crude extract while retaining enough DNA for amplification. A 7 mm^2^ (3-mm diameter) disc of Whatman No.1 paper was added to an *A*. *thaliana* leaf extract for one minute before transferring it to a tube containing wash buffer for one minute and finally transferring it to the PCR reaction tube, where it remained for the entire PCR process. The primers used in the PCR were designed to amplify a 262-bp fragment of the Arabidopsis G-protein gamma-1 subunit gene (*At3g63420*). No amplification occurred when either 1 μl of extract or a Whatman No.1 filter soaked in extract was directly added to the amplification mix (Fig 1C). However, briefly washing the extract-soaked filter paper once prior to using the filter directly in a PCR reaction was sufficient to remove amplification inhibitors while retaining the captured plant DNA (Fig 1C). Performing a second wash did not enhance or diminish the amplification efficiency.

### Method evaluation and application to different systems

**Fig 2:**
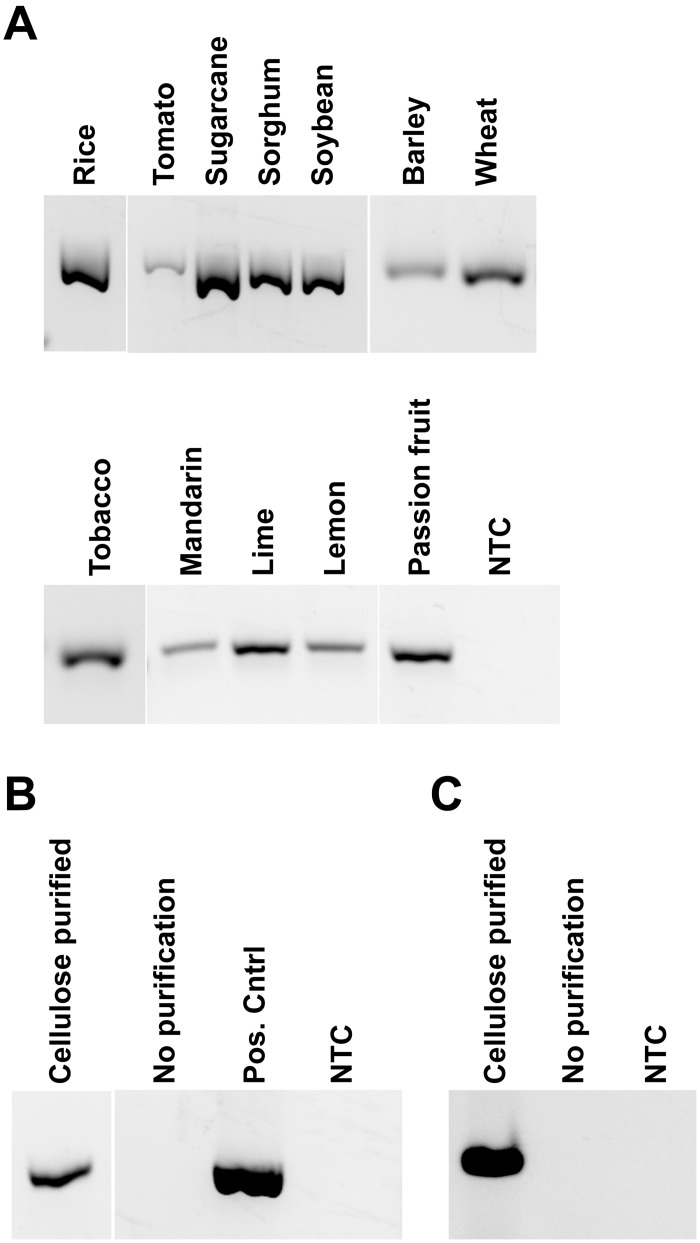
Cellulose-based paper is capable of purifying nucleic acids from a wide range of plant and animal tissues. (A) Genomic DNA from leaf tissues was extracted using the cellulose disc nucleic acid purification method. Universal primers designed against the 5.8S ribosomal RNA gene were used to amplify a product by PCR from each plant species with the exception of rice in which the betaine aldehyde dehydrogenase 2 (GenBank: KU308249.1) was amplified. (B) Human whole blood was diluted 1 in 5 in an extraction buffer containing proteinase K before using the cellulose disc method to purify genomic DNA in order to amplify a fragment of the BRAF gene (UniGene Hs.550061). 1 μl of each of the raw lysates was also added directly into separate PCR reactions. Purified Hela cells genomic DNA was used as a positive control. (C) Genomic DNA purified from a human melanoma cell line (LM-MEL-70) using the cellulose disc method was used to amplify a fragment of the 28S ribosomal gene. As a control, 1 μl of the raw lysate was added directly into a separate PCR reaction. NTCs involved adding 1 μl of water instead of DNA template. BRAF, human V-raf murine sarcoma viral oncogene homolog B1; NTC, no template control.

Although the method was successfully applied to the model plant species *A*. *thaliana*, our aim was to develop it further so that it would be suitable for DNA-based diagnostics of commercially important crops capable of deployment in challenging field environments. We successfully applied our cellulose-based DNA purification method to a number of agriculturally important species including wheat, barely, rice, soybean, tomato, and sugarcane (Fig 2A). The method was also successfully used to produce PCR-ready DNA from mature leaves of a number of citrus tree species (mandarin, lime, and lemon) (Fig 2A), which are notoriously difficult to extract nucleic acids from due to their high levels of lignin, phenolics, and polysaccharides [26].

Important human diseases such as HIV and Hepatitis can be diagnosed using nucleic acid-based tests from blood samples, although it is essential to remove several inhibitory compounds prior to DNA amplification [27]. For example, reagents such as proteinase K are used to help increase the efficiency of DNA extraction but can themselves inhibit DNA amplification. There are both commercial kits and published methods able to extract DNA from blood, but they require relatively extensive sample manipulation, which makes them suboptimal for PON applications and other limited resource environments (e.g., university classrooms and developing countries) [28–30]. We therefore tested whether cellulose filter paper is capable of purifying DNA from whole blood samples in order to amplify a fragment of the human V-raf murine sarcoma viral oncogene homolog B1 (BRAF) gene by PCR (Fig 2B). Cell lysis was achieved by diluting the blood samples 1 in 5 (v/v) in an extraction buffer containing proteinase K. Direct addition of the sample to the PCR reaction did not result in detectable amplification. In contrast, immersing the filter paper in the sample followed by a one-minute wash allowed amplification while removing inhibiting compounds from the sample, resulting in a clear amplification product. The cellulose disc method was also successfully used to amplify genomic DNA from melanoma cell line cultures while direct addition of lysate to the PCR reaction mix did not produce any amplicons (Fig 2C).

**Fig 3:**
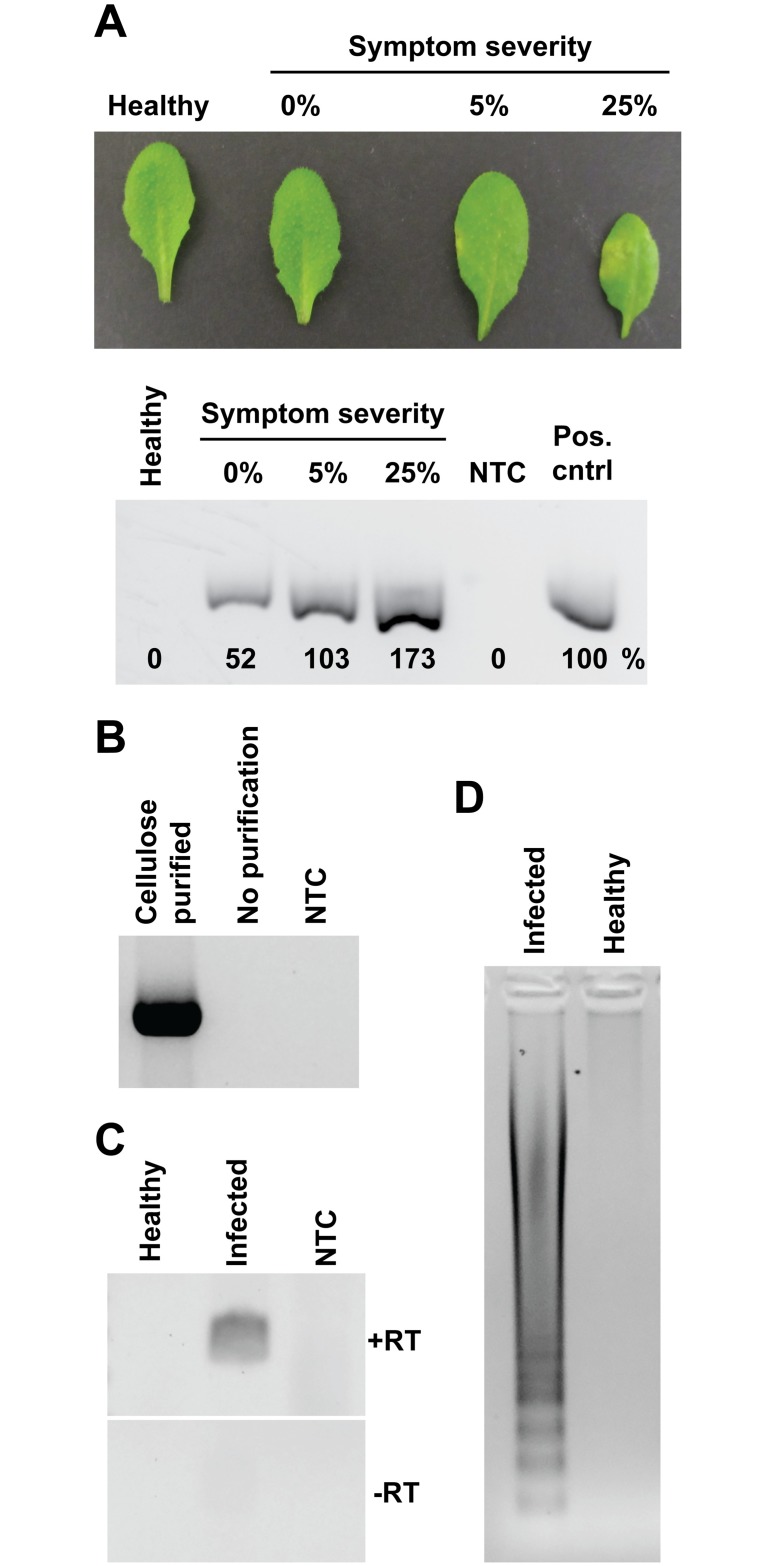
DNA and RNA extraction from plant and animal pathogens using cellulose-based paper. (A) DNA was purified from *P*. *syringae*-infected *Arabidopsis* leaves at different stages of infection using cellulose discs and a fragment of the *P*. *syringae* genome amplified by PCR. The band intensities achieved with cellulose discs relative to the positive control appear below each band. (B) A Whatman No.1 disc was used to purify DNA from a lung swab of a pig infected with *A*. *pleuropneumoniae* that had been placed in extraction buffer (see Material and methods). As a control, 1 μl of the raw lysate was added directly into a separate PCR reaction. (C) Whatman No.1 discs were used to purify nucleic acids from tomato plants infected with cucumber mosaic virus. The cellulose discs were added to RPA reactions with or without the presence of RT. NTCs involved adding 1 μl of water instead of DNA template. (D) Cellulose discs were used to purify nucleic acids from tomato plants that were either healthy or infected with cucumber mosaic virus and subsequently amplify them in a LAMP isothermal reaction. LAMP, loop-mediated amplification; NTC, no template control; RPA, recombinase polymerase amplification; RT, reverse transcriptase.

In our opinion, one of the ultimate applications for our method is in molecular diagnostic assays at the PON outside the modern laboratory, replacing the current labor-intensive procedures. To test the ability of the method to detect plant pathogens, we infected Arabidopsis plants with the bacterial pathogen *Pseudomonas syringae*. Our method successfully extracted and amplified pathogenic DNA even before the symptoms were visible to the human eye (Fig 3A). Furthermore, as the size of the cellulose disc and the tissue to extraction buffer ratio were kept constant between samples, the disease progression could be quantified by PCR as the band intensities increase with increasing disease severity. The cellulose disc method was also successfully used in the detection of an animal bacterial pathogen, *Actinobacillus pleuropneumoniae*, in a lung swab from an infected pig (Fig 3B). Finally, we tested if the method could be used for the extraction of RNA as many plant and animal pathogens have RNA genomes. Tomato plants infected with cucumber mosaic virus (CMV) were tested using the filter paper DNA extraction method without any modifications. In this case, we used recombinase polymerase amplification (RPA) (isothermal) with the addition of reverse transcriptase to the reaction mix in order to perform reverse transcription and amplification simultaneously in a single tube. An amplification product was obtained in reactions containing reverse transcriptase, while no amplification was observed on uninfected samples or reactions lacking reverse transcriptase (Fig 3C). The cellulose disc method also works in conjunction with other isothermal methods including Loop-mediated amplification (LAMP), which detected the CMV RNA without requiring a reverse transcriptase due to the intrinsic reverse transcriptase activity of the Bst 2.0 enzyme [31] (Fig 3D).

### DNA capture and release

To explore the mechanism behind DNA capture by Whatman No.1 paper, we examined whether we could replace it in our simple extraction method with different types of solid supports. Our results show that other cellulose-based papers, including a common hand drying paper towel (Scott brand “Optimum towel”) can be used to purify crude plant extracts (S2A Fig). However, not all cellulose papers can be used to purify nucleic acids as common photocopy paper, either bleached or unbleached, failed to amplify a product. We successfully used nylon membranes (Qiagen Qiabrane, Amersham Hybond-N) to purify DNA from the plant extract revealing that this method is not limited to cellulose-based supports. Positively charged supports failed to produce amplicons, independently of whether they were nylon- or cellulose-based (Amersham Hybond-N+, Qiabrane nylon plus, Hybond-C extra (nitrocellulose) and DEAE cellulose). This result indicates that materials that are ideal for DNA capture are not necessarily adequate for use in DNA amplification.

We determined whether the amount of the cellulose used in the DNA extraction has an effect on the amplification yield. After using 1, 2, or 3 discs in the extraction procedure, we observed an inverse relationship between the number of cellulose or nylon discs used and the amount of DNA amplified. It is plausible that increasing amounts of cellulose could result in greater sequestration of primers, deoxynucleotide triphosphates (dNTPs) or other reagents in the amplification reaction. Consistent with this, Scott paper towels which have 24% less cellulose by weight compared to Whatman No.1 resulted in stronger amplification when an equal number of discs were used (S2B Fig).

**Fig 4:**
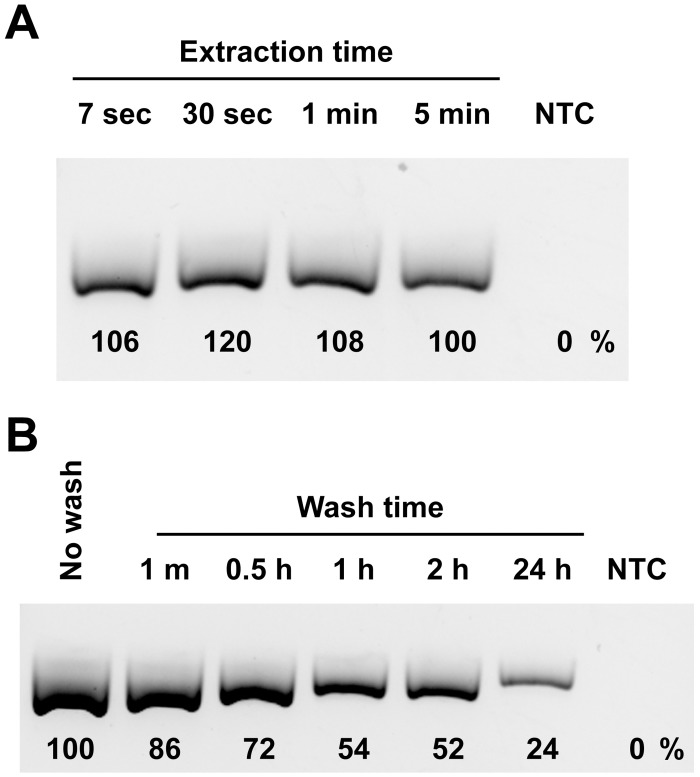
Cellulose-based paper rapidly binds DNA but releases it slowly. (A) Whatman No.1 discs were exposed to a 1 ng/μl purified *Arabidopsis* genomic DNA solution for different amounts of time before washing for one minute and transferring to a PCR reaction. The band intensities achieved with cellulose discs relative to the 5 minute sample appear below each band. (B) 10 ng purified *Arabidopsis* genomic DNA was pipetted onto Whatman No.1 discs which were then washed in 10 ml of water with gentle agitation for different lengths of time prior to transferring the disc to a PCR reaction. The band intensities achieved with cellulose discs relative to the no wash sample appear below each band. NTC, no template control.

We hypothesised that the mechanism underlying our rapid purification method is based on differences in the kinetics of nucleic acid binding to, and release from, the cellulose. In our model, nucleic acids are able to rapidly bind to the cellulose fibres but are released at a much slower rate. Of note, other components present in the sample extracts, such as amplification inhibitors, either do not bind to the cellulose or are rapidly released and subsequently removed from the cellulose matrix during the brief washing step. As predicted, we found that DNA binds to Whatman No.1 and rapidly reaches an equilibrium with the surrounding liquid (Fig 4A). Briefly exposing the cellulose disc to a 1 ng/μl DNA solution for seven seconds, followed by a wash step, resulted in strong amplification that could not be improved by longer incubation times. In contrast, during the washing step, we found that DNA was released from the cellulose at a relatively slow rate (Fig 4B). Whatman No.1 discs with 10 ng of purified genomic DNA directly added to them were washed for varying lengths of time by gently rocking in 10 ml of water before being dropped in the PCR amplification mix. As expected, the disc that was not washed, and therefore contained the entire DNA sample, gave the highest band intensity after amplification. Also, consistent with our hypothesis, Whatman No.1 washed for up to 24 hours still retained enough DNA to give a positive amplification product. Most importantly, a brief one-minute wash did not greatly affect the amount of DNA retained in the filter as can be seen by the similar intensity of the amplification bands when compared to the no wash control.

**Fig 5:**
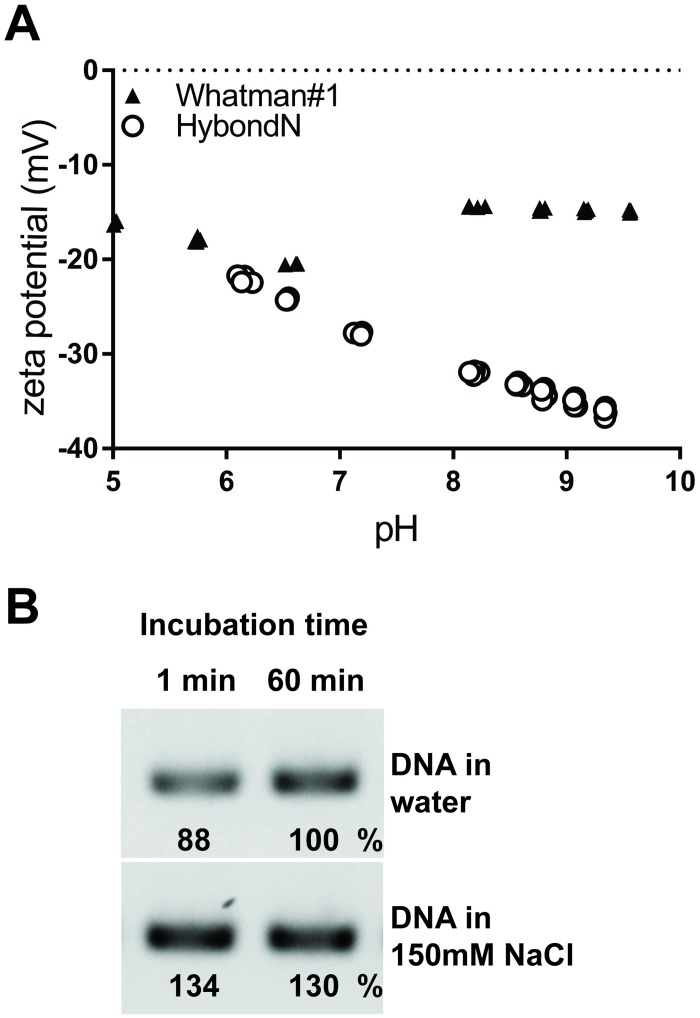
Salts enhance DNA binding to cellulose-based paper. (A) The zeta potential of Whatman No.1 (black triangles) and Hybond N (open circles) was measured across a range of pHs. (B) Whatman No.1 discs were incubated in purified *Arabidopsis* genomic DNA (1 ng/μl) dissolved in water or in 150 mM NaCl. DNA solution was removed from discs by spinning, and the discs were added to a PCR amplification. The band intensities achieved with cellulose discs relative to the 60 minute sample in water appear below each band.

To better understand the nature of the interaction between the nucleic acids and the Whatman No.1, we examined the paper’s surface charge by measuring the streaming potential. We found that the surface of Whatman No.1 has a negative zeta potential that remains relatively constant across the range of pHs tested (pH 5 to pH 10) (Fig 5A, S1 Data). Similarly, the nylon-based Hybond N membrane, which can also be used to purify nucleic acids, also shows a net negative zeta potential that decreases in absolute value with increasing pH. These results are consistent with previous studies that reported a negative surface charge for cellulose and nylon due to the presence of acidic groups, such as carboxyl groups, on their surface [32,33]. As DNA also carries a net negative charge, largely due to its electronegative phosphate backbone, the like charges between the DNA and the Whatman No.1 surface will result in a repulsive force that will hinder DNA binding. We therefore predicted that the addition of salt could increase the binding of DNA to the Whatman No.1 paper by counteracting the electrostatic repulsion. Our results support our hypothesis as we observed a 30%–46% increase in DNA amplification from samples diluted in 150 mM NaCl compared to those diluted in water (Fig 5B).

### Pipette-free nucleic acid purification

**Fig 6:**
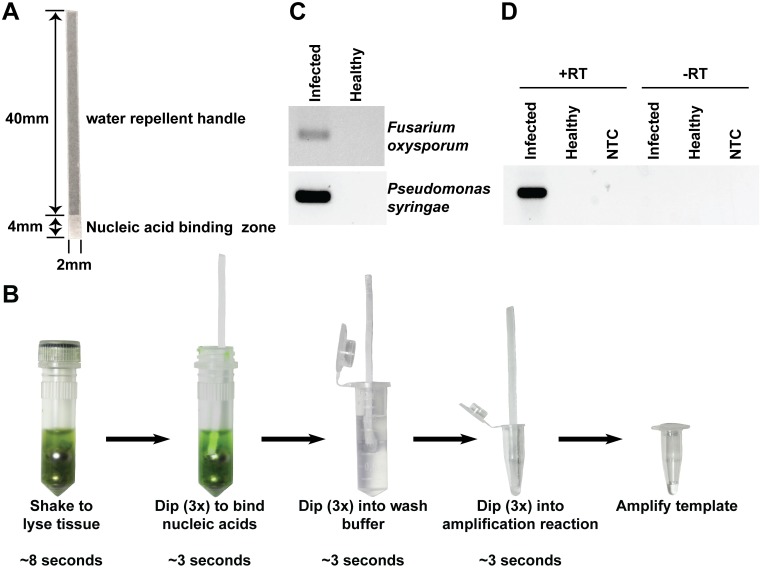
Overview of the dipstick based nucleic acid purification. (A) The cellulose dipstick consists of a 2x40 mm wax impregnated handle and a 2x4 mm nucleic acid binding zone. (B) An overview of the dipstick-based purification method in which tissue is homogenised by shaking it in a tube containing ball bearings and an appropriate extraction buffer. The dipstick is used to bind the nucleic acids by dipping it three times into the homogenate, washed by dipping it three times into a wash buffer, and eluted by dipping it three times in the amplification reaction mix. (C) Nucleic acids were purified using the cellulose dipstick method from *Arabidopsis* leaves infected with *F*. *oxysporum* f.sp. *conglutinans* (upper image) or *P*. *syringae* (lower image) and eluted into PCR reaction mixes containing pathogen-specific primers. (D) Nucleic acids were purified from tomato leaves infected with Cucumber mosaic virus using the cellulose dipstick method. The purified DNA was eluted directly into PCR amplification reaction mixes with (+RT) or without (-RT) AMV reverse transcriptase. NTCs involved adding 1 μl of water instead of using dipstick-purified nucleic acids. AMV, Avian myeloblastosis virus; NTC, no template control; RT, reverse transcriptase.

Based on the information gained in this study, we aimed to further simplify the nucleic acid extraction method by using a cellulose-based dipstick in order to streamline the handling and eliminate the need to transfer the cellulose disc between tubes. To this end, we designed dipsticks made from Whatman No.1 with a small 8 mm^2^ DNA binding surface and a long water repellent handle made by impregnating the filter paper with paraplast wax (Fig 6A). Using these dipsticks, we developed an improved method in which all reagents can be prepared in advance and stored for a long period of time at room temperature. When needed, a nucleic acid extraction can be performed rapidly in three easy steps and less than 30 seconds without a pipette or any electrical device (Fig 6B, S1 Movie). Tissue is first homogenised in a tube containing the appropriate lysis buffer and ball bearings to help macerate the tissue. The cellulose dipstick is used to capture nucleic acids by dipping it into the lysate three times. Contaminants are removed from the dipstick by dipping it up and down in a wash solution three times. Finally, the bound nucleic acids are eluted from the cellulose by dipping the dipstick directly into the amplification mix three times. Using this method, we have successfully demonstrated that it is possible to rapidly purify DNA from plant leaves infected with the fungus *Fusarium oxysporum* or the bacteria *P*. *syringae* (Fig 6C). Additionally, this method works equally well in extracting viral RNA from plant leaves that is suitable for use in reverse transcription PCR amplification (Fig 6D).

**Fig 7:**
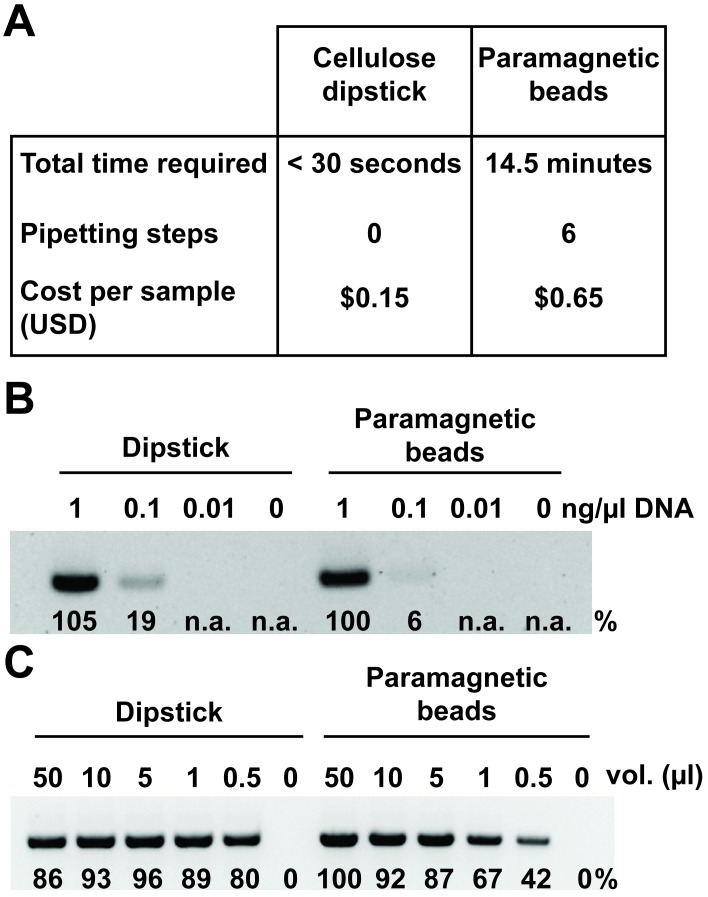
Cellulose dipsticks outperform a commercially available nucleic acid purification system. (A) The time required, number of pipetting steps involved, and the costs of all consumables—including tubes and pipette tips—were calculated for purification of nucleic acids from *Arabidopsis* leaf tissue using either the cellulose dipstick or Agencourt AMPure paramagnetic beads. All solutions that could be prepared in advance, including lysis and wash buffers, were made and prealiquoted. The time and pipetting involved in the preparation of these solutions was not added to the tallies in the table. (B) Purified *Arabidopsis* DNA at different concentrations was a captured, washed, and eluted using either the cellulose dipsticks or AMPure paramagnetic beads (Beckman Coulter). The eluted DNA was used in a PCR reaction with using primers designed for the G-protein gamma subunit 1 gene. The band intensities relative to the 1 ng/μl paramagnetic bead sample appear below each band. (C) Different volumes of an *Arabidopsis* leaf extract were captured, washed, and eluted using either the cellulose dipsticks or AMPure paramagnetic beads and subsequently amplified in a PCR reaction as described above. The band intensities relative to the 50 μl paramagnetic bead sample appear below each band. n.a., no amplification; USD, United States Dollar.

To validate our newly developed nucleic acid purification method, we compared it with a popular commercial rapid paramagnetic bead DNA extraction method (Beckman coulter, AMPure). We found that our method can purify amplifiable DNA significantly faster: under 30 seconds for our method versus 14.5 minutes for AMPure purification when following the manufacturers’ recommended instructions (Fig 7A). Importantly, the method achieves this speed and simplicity without the need for any pipetting. Our method is significantly cheaper, with consumables costing four times less than those required by the AMPure system and does not require the initial investment of USD $685–$876 for the specialized magnet plate. The sensitivity of our method was comparable to the commercial system, as they could both extract amplifiable DNA from initial concentrations of 0.1 ng/μl genomic DNA and above (Fig 7B). In some experimental settings, there is a limited supply of sample tissue and it is therefore critical to be able to extract DNA from small volumes of tissue extract. We found that our method was again comparable the commercial system in its ability to purify nucleic acids from tissue extract volumes as low as 0.5 μl (Fig 7C).

## Discussion

The development of new molecular technologies for PON diagnostics is now proceeding and an unprecedented pace. Nucleic acid-based (molecular) assays offer greater sensitivity, specificity and speed over other technologies including enzyme-linked immunosorbent assay (ELISA), lateral flow strips and cell culture/analysis [1,34,35]. As such, molecular assays have the potential to revolutionize the early detection and continual monitoring of human, plant, and animal diseases. However, a major bottleneck in molecular diagnostics is that they rely on nucleic acid purification, which is a relatively time consuming and laborious procedure that is not easily performed outside the modern laboratory environment (e.g., field-based testing) [36–38].

Our study reveals that a small (approximately 8 mm^2^) piece of cellulose-based paper is capable of purifying nucleic acids away from inhibitors in a wide range of plant, animal, and microbial samples including whole blood and mature tree leaves (Figs 2 and 3). To make the method suitable for field-based testing, we incorporated the knowledge gained in this study to create a cellulose-paper-based dipstick that can bind, wash, and elute purified nucleic acids in under 30 seconds without requiring any pipetting or electrical equipment (S1 Movie). A significant advantage of the method presented here is that the amount of nucleic acid transferred to the amplification reaction will be similar between samples of the same type because the size of the DNA binding surface on the cellulose dipstick remains constant. Furthermore, the system can be fine-tuned by altering the size of the DNA binding surface in the dipstick, thus optimising the amount of nucleic acid transferred for downstream applications. This is an important feature as it provides flexibility to adapt the method to different tissues (e.g., plant leaves, blood, and saliva) depending on the intended application. We have demonstrated that, despite its speed and simplicity, this method is comparable to a commercially available nucleic acid purification method in its ability to prepare amplification-ready template nucleic acids (Fig 7). Therefore, we believe that the speed, simplicity, and universality of this method makes it an attractive option in a broad range of diagnostic applications both within and outside the laboratory environment.

As this study was focused on creating a simple, pipette-free nucleic acid purification method and not a complete molecular diagnostic system, a mains powered thermocycler was used for most reactions, which is obviously not ideal for research outside the laboratory environment. We have demonstrated that our method can be coupled with isothermal DNA amplification technology (Fig 3C and 3D), which have previously been successfully performed using simple portable devices that generate heat using either a small battery or chemical heat pad [39–42]. Therefore, it is conceivable that a simple molecular diagnostic assay that requires minimal equipment and no pipetting can be created by coupling our dipstick nucleic acid purification system with isothermal DNA amplification and equipment-free naked eye visualisation methods [16,21,43–48]. Such a system would not only be ideal for field-based PON diagnostics but would make molecular-based testing more accessible to a greater spectrum of the community including those in university and high school classrooms, farmers, biosecurity, and remote/resource-limited environments.

Overall cost, including the equipment required to perform the procedure, is a major determinant in the likelihood of broad scale adoption of new diagnostic technologies [49,50]. The cellulose dipstick purification method described here significantly increases the affordability of nucleic acid purification, with the price per sample being $US 0.15 including plasticware and reagents, with the ball bearings being the major contributor to the final price (Fig 7A). If the ball bearings used to homogenize the tissue are washed and reused, the cost can be further reduced to just $US 0.06 per sample. Whatman No.1 paper is cheap and easy to obtain but not absolutely necessary as common paper towels proved to be equally efficient, providing an even cheaper alternative.

The dipstick-purification system presented here has numerous advantages over the vast array of commercially available and published nucleic acid extraction procedures. The most obvious advantage is that it is significantly faster and has fewer steps than common liquid-based DNA extraction methods (e.g., phenol/chloroform, hexadecyltrimethylammonium bromide [CTAB], or guanidinium salts) or solid-phase DNA extraction methods involving silica or paramagnetic beads (Fig 7) [5,9,51–53]. The method is more closely related to a number of recently developed DNA extraction methods that utilize commercially available filters, including the silica-based Whatman Fusion 5 and the cellulose-based Whatman FTA cards [12,14,15,54]. However, our method is much simpler and faster than any of the available membrane-based procedures. For example, FTA cards contain chemicals that lyse cells and protect the DNA from degradation and have been used for over a decade as a means to store and preserve DNA samples before processing [55–57]. These chemicals are inhibitory to DNA amplification and therefore must be removed through a number of washing and drying steps [14] before the DNA can be amplified from the FTA card. Additionally, unlike the 2-minute Fusion-5-based purification method, which can only capture DNA [13,15], our method using Whatman No.1 can also be used to extract RNA suitable for reverse-transcription and subsequent DNA amplification (Figs 3C, 3D and 6D).

The use of cellulose for DNA purification is commercially available and is claimed to have improved performance over silica-based DNA purification methods [58,59]. DNA purification using cellulose has been previously reported to be achieved by coaggregating or adsorbing the DNA to the cellulose in the presence of various chemicals, including chaotropic salts [60], ethanol [23,61], and high salt concentrations and/or crowding agents such as polyethylene glycol [11,62,63], which destabilise the DNA structure and facilitate its interaction with the cellulose fibres. In these systems, water or a low salt solution is then required to elute the DNA from the cellulose. In contrast, we found that cellulose can capture DNA in pure water (Fig 5B) and, moreover, retain the DNA in the presence of a large volume of water for over 24 hours (Fig 4B). Furthermore, we demonstrated that the method is not dependent on a specific buffer but could successfully purify nucleic acids from crude extracts with either guanidine hydrochloride-based (e.g,. Figs 2B and 3B), SDS-based (Fig 6C and 6D), or Tween 20-based (e.g., Fig 1C) extraction buffers. Although the exact mechanism of nucleic acid binding remains unclear, we observed that the amount of nucleic acid bound can be enhanced in the presence of salts (Fig 5B). This is likely due to the neutralization of the negative charges on the surfaces of both the cellulose and nucleic acids thereby eliminating the repellent electrostatic forces between them.

Our cellulose-based method for nucleic acid purification takes advantage of four key cellulose characteristics. First, cellulose paper is capable of rapidly absorbing a relatively large amount of DNA and/or RNA relative to its mass through capillary action [64]. Second, nucleic acids are either rapidly entrapped by, or bind to, the cellulose fibers (Fig 4A). Third, a sufficient amount of nucleic acid is retained on the cellulose even after extended incubation in a large volume of water, while inhibitors including Proteinase K and cellulosic and phenolic compounds are rapidly eluted (Figs 1C, 2B and 4B). Lastly, unlike positively charged membranes (S2A Fig), cellulose enables rapid elution of a sufficient quantity of bound nucleic acids into the amplification mix (Fig 6C and 6D). This rapid elution from the cellulose is likely catalysed by dNTPs present in the amplification mix as has been reported for other systems [65]. Collectively, these characteristics make cellulose an ideal material for a rapid and simple nucleic acid purification system that easily separates unwanted contaminants and inhibitors away from nucleic acids while also transferring a reproducible amount of nucleic acids into the amplification mix.

We have presented here a simple and rapid technique that allows researchers to obtain nucleic acid at a suitable purity and concentration for DNA amplification. We have reduced a complicated process to three simple steps that do not require any specialized equipment (e.g., pipettes or centrifuges) and takes less than 30 seconds to perform (S1 Movie). As such, the simplicity and speed of this method as well as the low cost and availability of suitable dipstick materials means that nucleic acid extraction is now more accessible and affordable for a wide variety of people and applications both inside and outside the laboratory environment.

## Materials and methods

### Ethics statement

Human research ethics approval was obtained from The University of Queensland Institutional Human Research Ethics Committee (Approval No. 2004000047). Animal research ethics approval was obtained from The University of Queensland Animal Ethics Committee (Approval No. AE14701).

### Plants, animals, and microbes

Plant materials used in this study included *A*. *thaliana* ecotype Columbia, tobacco (*Nicotiana benthamiana*), tomato (*Solanum lycopersicum* cv. MicroTom), sugarcane (*Saccharum officinarum* cv. Q208), sorghum (*Sorghum biocolor* cv. IS8525), soybean (*Glycine max* cv. Bunya), rice (*Oryza sativa* cv. Topaz), barley (*Hordeum vulgare* line 21:ZIB14), wheat (*Triticum aestivum* line S19-49), mandarin (*Citrus reticulata*), lime (*C*. *aurantiifolia*), lemon (*C*. *limon*), passion fruit (*Passiflora edulis*). Diseased plant materials included *A*. *thaliana* leaf tissue infected with *P*. *syringae* pv tomato strain DC3000 or *F*. *oxysporum* f.sp. *conglutinans*, and tomato leaf tissue infected with cucumber mosaic virus. Human samples included melanoma cell line LM-MEL-70 and blood. Diseased animal material was harvested by a swab from a pig lung infected with *Act*. *pleuropneumoniae*.

### *A*. *thaliana* DNA purification

To investigate nucleic acid—cellulose interaction, *A*. *thaliana* (ecotype Columbia) DNA was extracted by modified CTAB DNA extraction [66]. *A*. *thaliana* leaves were finely ground using liquid nitrogen, and approximately 100 mg of leaf powder was mixed with 500 μl of extraction buffer (2% w/v CTAB, 1.42 M NaCl, 20 mM EDTA, 100 mM Tris HCl [pH 8.0], 1% w/v polyvinylyrilodone [PVP 40]) that was preheated 60°C. After 45 minutes at 60°C, 500 μl of chilled chloroform: isoamyl alcohol (24:1, v/v) was added into the mixture and rocked gently at room temperature for 15 minutes, followed by centrifugation at 15,000 xg for 10 minutes. 200 μl supernatant was transferred to a new tube and mixed gently with 400 μl chilled ethanol. After incubation at −20°C for 1 hour, the sample was centrifuged at 15,000 xg for 10 min to pellet the DNA. The pellet was washed with 80% ethanol, followed by 100% ethanol. DNA was suspended with 100 μl of H_2_O and 50 μg RNase A and followed by incubation at 37°C for 20 minutes to degrade RNA. 10 μl of 3 M sodium acetate and 100 μl of isopropanol were added into sample and incubated at -20°C for 10 minutes. DNA was pelleted by centrifugation at 15,000 xg for 2 minutes and washed with 80% ethanol. After air drying, the DNA was suspended with 50 μl of H_2_O and quantified with NanoDrop ND-1000 spectrophotometer.

### DNA binding to chemically modified cellulose

A number of chemicals with potential DNA binding capability were selected including spermine, PVP 40 and the cationic polymers: PEI, dopamine, 3-aminopropyl trimethoxysilane (APTMS), and chitosan. Solutions were made containing the chemicals at either 1.25% (w/v) (Chitosan, APTMS, PEI) or 2.5% (w/v) (dopamine, spermine, PVP-40). 1 μl of each solution was carefully added to two 70-mm Whatman No.1 discs approximately 10 mm from the centre of the disc. The chemicals were allowed to fully dry onto the paper before viewing the filter under UV light to assess the amount of fluorescence each chemical induces in the absence of DNA. 150 μl of 500 ng/μl salmon sperm DNA (Sigma) labelled with 0.5% (v/v) GelRed (Biotum) and buffered in either 50 mM MES (pH 5) or 50 mM Tris (pH 8.5) was added to the centre of each Whatman No.1 disc. After approximately 5 minutes, the movement of DNA by capillary action had stopped and the cellulose disc was viewed under UV light. DNA binding by the chemical was indicated by brighter fluorescence over the background.

### DNA binding and PCR amplification with chitosan-treated cellulose

3-mm diameter discs were cut from a piece of Whatman No.1 using a hole puncher, to which 1 μl of 1.25% (w/v) chitosan in 50 mM acetic acid was added. After the chitosan had dried, the cellulose discs were incubated for one minute in 20 μl of 100 pg/μl purified Arabidopsis DNA buffered with 10 mM MES (pH5). The cellulose pieces were transferred into a tube containing 200 μL of 10 mM MES (pH5) or 10 mM Tris (pH8.5), briefly agitated by pipetting up and down and then incubated for one minute prior to transferring into a PCR reaction. The results of the amplification reaction were visualised by agarose gel electrophoresis.

### Cellulose disc nucleic acid purification

For nucleic acid purification from plant tissues, 5–10 mg of leaf tissue was ground in a 1.5-ml tube with a plastic pestle in presence of 50 μl extraction buffer #1 (50 mM Tris [pH 8.0], 150 mM NaCl, 2% PVP, 1% Tween-20) for approximately 30 seconds. A 3-mm diameter disc was cut from a piece of Whatman No.1 using a hole puncher and transferred into the tissue extract for a minimum of three seconds. The disc was then transferred to 200 μl of wash buffer (10 mM Tris [pH 8.0], 0.1% Tween-20) using a pipette tip to remove contaminants including amplification inhibitors. After one minute, the disc containing nucleic acid was then transferred into an amplification reaction using a pipette tip. For RNA purification from CMV-infected tomato leaves, samples were prepared as described above with the exception that 50 μl of extraction buffer #2 (800 mM guanidine hydrochloride, 50 mM Tris [pH 8], 0.5% Triton X100, 1% Tween-20) was used to lyse the samples instead of extraction buffer #1.

For DNA purification from blood, samples were mixed with four volumes of extraction buffer #2 with the addition of 40 μg/ml Proteinase K to aid DNA extraction. For DNA purification from human cell lines, 100 μl of cultured LM-MEL-70 cells were gently spun down (500 xg, 5 minutes), washed in 1XPBS buffer, and lysed by adding 200 μl extraction buffer 2 and vortexing for 10 seconds. A 3 mm Whatman No.1 disc was incubated in cell lysate for one minute. The cellulose disc was transferred to 200 μl of wash buffer for one minute before transferring to PCR reaction mix.

For DNA extraction from the pig lung, the surface of the lung was sterilised by cauterization with a hot spatula. An incision was made in the cauterized area of the lung and a cotton swab inserted into the inner lung tissue and then dropped into 500 μl of extraction buffer #3 (1.5 M guanidine hydrochloride, 50 mM Tris (pH 8), 100 mM NaCl, 5 mM EDTA, 1% Tween-20). A 3 mm Whatman No.1 disc was incubated in the extract for one minute. The cellulose disc was transferred to 200 μl of wash buffer for one minute before transferring to PCR reaction mix.

### Characterising DNA—Cellulose interaction

Unless otherwise stated, experiments that investigated DNA binding or release from cellulose were performed as follows. A hole puncher was used to generate 3-mm diameter cellulose discs from a piece of Whatman No.1 filter paper. Purified Arabidopsis genomic DNA was pipetted onto individual cellulose discs that were subsequently transferred into a tube containing 200 μl of wash buffer (10 mM Tris (pH 8.0), 0.1% Tween-20) using a pipette tip. After one minute in wash buffer, the cellulose discs were transferred into a PCR amplification reaction and the amplifications including control reactions, performed in a thermocycler as described below. The same method was used to compare the performance of various commercially available membranes including Whatman No.1 and No.4 (GE Life sciences), Whatman DEAE-cellulose (GE Life sciences), filter paper (Invitrogen), blotting filter paper (Immobilon), FL PVSF (Immobilon), Hybond N and N+ (Amersham), nylon and nylon plus (Qiabrane), hybond-C extra (Amersham), bleached and unbleached photocopy paper (Australian paper), Scott Optimum hand towel (Kimberly-Clark).

For analysis of DNA release from the cellulose, the disc containing 10 ng of purified DNA was then transferred into 10 mL of wash buffer (10 mM Tris [pH 8.0], 0.1% Tween-20) and placed on a Mini rocker (Bio-Rad) and agitated gently for 0 to 24 hours. After agitation, the cellulose disc was transferred into a PCR reaction and the amplification performed in a thermocycler.

### Streaming potential measurements

An electrokinetic analyzer for solid surface analysis (SurPASS, Anton Paar) was used to determine surface charge of Whatman No.1 and Hybond-N filters. Two pieces of each filter (approximately 15 mm x 25 mm) were fixed on opposite sample holders by double-sided adhesive tape with an approximately 100 μm gap between each other. The streaming potential resulting from pressure-driven 10 mM NaCl flowing through the gap was measured. The pH of the solution was automatically altered by the addition of 100 mM NaOH. The streaming potential of paper as well as pH of the NaCl solution was recorded at set intervals.

### Dipstick nucleic acid purification and subsequent amplification

Dipsticks were created by dipping half of a Whatman No.1 filter into molten wax (Paraplast Plus, Fluka) to create a region that is impervious to water. After the wax had set, the partially wax-coated filter paper was cut into a 44 mm-wide rectangle, of which approximately 40 mm was coated in wax and 4 mm was uncoated. This rectangle was then cut into approximately 2 mm-wide strips to create dipsticks with a 2x4 mm nucleic acid binding area and a 2x40 mm handle.

For nucleic acid purification using the dipsticks, leaf tissue (approximately 200 mm^2^) was added to a 2 mL tube containing 500 μl cell lysis buffer (20 mM Tris [pH 8.0], 25 mM NaCl, 2.5 mM EDTA, 0.05% SDS), and two ball bearings. The plant tissue was macerated by shaking the tube for approximately eight seconds. The dipstick was dipped into extract to bind nucleic acids then dipped into 1.75 mL of wash buffer (10 mM Tris [pH 8.0], 0.1% Tween-20) and then finally the bound nucleic acids were eluted by dipping the dipstick directly into amplification reaction. Each time the dipstick was dipped up and down in each solution three times, taking approximately three seconds. After elution, the dipstick was discarded and the DNA amplification reaction transferred to a thermocycler.

### Magnetic beads nucleic acid extraction

Agencout AMPure XP PCR Purification kit (Beckman Coulter) was used to purify DNA following the manufacturer’s recommendations. Briefly, one volume of sample was mixed with 1.8 volumes of paramagnetic particles. The mixture was incubated at room temperature for five minutes and then placed onto magnetic plate (Life technologies) for two minutes to pull down DNA-bound paramagnetic particles. After supernatant was removed, paramagnetic particles were washed twice with 70% ethanol. After the 70% ethanol from the last wash was removed, the paramagnetic beads were air dried for five minutes. The bound DNA was eluted by resuspending the particles in 40 μl of water and incubating at room temperature for one minute before pulling the particles down to the bottom of the tube by magnet. The supernatant containing purified DNA was transferred to a new tube and was later used as template DNA in PCR amplifications.

### Nucleic acid amplification

Nucleic acid amplification was performed by either PCR, LAMP, or RPA. For PCR amplification, 15 μl reactions were performed using 7.5 μl of GoTaq Green Master Mix (Promega), 15 pmol of both forward and reverse primers (S1 Table), and template DNA. Unless otherwise stated, PCR cycling parameters were as follows: 95°C for two minutes, 35 cycles of 95°C for 20 seconds, 55–60°C for 20 seconds, 72°C for 40 seconds, followed by final extension of 72°C for one minute. Quantification of PCR band intensities was performed by using the “Analyse: Gels” function within ImageJ (V1.48) software [67].

For reactions involving RPA, either the TwistAmp Basic RPA or Basic RT-RPA kits (Twist DX) were used as per manufacturer’s recommendations. Briefly, each RPA pellet was resuspended with 29.5 μl of rehydration buffer and 0.8 μM of both forward and reverse primers (S1 Table). The mix was then aliquoted evenly into four 0.2 ml tubes into which the template DNA, water, and 0.625 μl of 280 mM magnesium acetate are added to make a final volume of 12.5 μl. RPA or RT-RPA reactions were performed at 37°C or 42°C for 20 minutes, respectively, and the results visualised by agarose gel electrophoresis.

For reactions involving LAMP, cellulose discs containing extracted nucleic acids were added to 15 μl reactions containing: 20 mM Tris (pH 8.8), 10 mM (NH_4_)_2_SO_4_, 50 mM KCl, 0.1% (v/v) Tween-20, 0.8 M betaine, 8 mM MgSO_4_, 1.2 mM dNTPs, 4.8 U Bst2.0 warmstart (NEB Biolabs, USA), 0.8 μM of FIP and BIP primers, and 0.2 μM of F3 and B3 primers. Reactions were incubated at 63°C for 50 minutes, followed by a five-minute incubation at 80°C to denature the enzyme.

## Supporting information

S1 Fig Chemicals that are good for DNA capture aren’t necessarily good for DNA amplification. (A) GelRed-labelled salmon sperm DNA in pH5 (left image) or pH 8.5 (right image) buffer was added to the center of a Whatman No.1 filter disc on which the chemicals: 1.25% chitosan (1), 2.5% dopamine (2), 2.5% spermine (3), 2.5% polyvinylpyriliodone (4), 1.25% polyethylenimine (5), and 3-Aminopropyl-trimethoxysilane (6) had been spotted. The filters were viewed under UV light before (upper images) and after (lower images) DNA addition. (B) 3-mm diameter discs of Whatman No.1 paper that had been treated with or without 1.25% chitosan were incubated in *A*. *thaliana* genomic DNA for one minute, then washed in pH 5 or pH 8.5 buffer for one minute and then transferred to a PCR mix for amplification. 1 μl of water was used in place of the cellulose disc in the NTC. NTC, no template control.
(TIF)

S2 Fig DNA extraction can be achieved by a variety of solid support matrices. (A) Identical size fragments of a variety of sources were used to purify nucleic acids from an *Arabidopsis* leaf extract. The extracted nucleic acids were used for PCR amplification using primers designed for the G-protein gamma subunit 1 gene (*At3g63420*). (B) One, two, or three discs (3-mm diameter) of Whatman No.1, Hybond N or Scott-brand paper towel were incubated in purified *Arabidopsis* DNA, washed, and then used in a PCR reaction using primers designed for the G-protein gamma subunit 1 gene.
(TIF)

S1 Movie Video of the cellulose dipstick nucleic acid purification protocol applied to a plant leaf sample. A leaf is excised from a plant and placed into a tube containing lysis buffer and two ball bearings. The tissue was macerated by shaking the tube for approximately 8 seconds. The cellulose dipstick is dipped into the lysate to bind nucleic acids, then dipped in wash buffer to remove contaminants and finally dipped into amplification mix to elute the nucleic acids.
(MP4)

S1 Data Cellulose and Hybond N are negatively charged. The measured zeta potentials of Whatman No.1 and Hybond N across a pH range.
(XLSX)

S1 Table Oligonucleotide sequences. The names, sequences, target species, and source of each oligonucleotide used in this study.
(DOCX)

## Abbreviations

APTMS: 3-aminopropyl trimethoxysilane
BRAF: V-raf murine sarcoma viral oncogene homolog B1
CMV: cucumber mosaic virus
CTAB: hexadecyltrimethylammonium bromide
dNTP: deoxynucleotide triphosphate
ELISA: enzyme-linked immunosorbent assay
FTA: Flinders Technology Associates
LAMP: loop-mediated amplification
NTC: no template control
PEI: polyethylenimine
PON: point-of-need
PVP 40: polyvinylyrilodone
QTL: qualitative trait loci
RPA: recombinase polymerase amplification
