# Correction: Nucleic acid purification from plants, animals and microbes in under 30 seconds

**DOI:** 10.1371/journal.pbio.1002630

**Published:** 2018-05-07

**Authors:** Yiping Zou, Michael Glenn Mason, Yuling Wang, Eugene Wee, Conny Turni, Patrick J. Blackall, Matt Trau, Jose Ramon Botella

The Competing interests statement for this paper is incorrect. The correct statement is: The research generated in this manuscript was used to file a provisional patent application in Australia to protect any possible intellectual property arising from this work. Provisional patent application number: 2017901487. Title: SIMPLE DNA EXTRACTION. Applicant: The University of Queensland. Filing date: April 24, 2017. The authors have not licensed or sold their technology to any company and it is not being commercialized by them.
