# Supplementary figures and images for: Nucleic acid purification from plants, animals and microbes in under 30 seconds
Source: PLoS Biol. 2017 Nov 21;15(11):e2003916. doi: 10.1371/journal.pbio.2003916 (PMC5697807; doi:10.1371/journal.pbio.2003916)

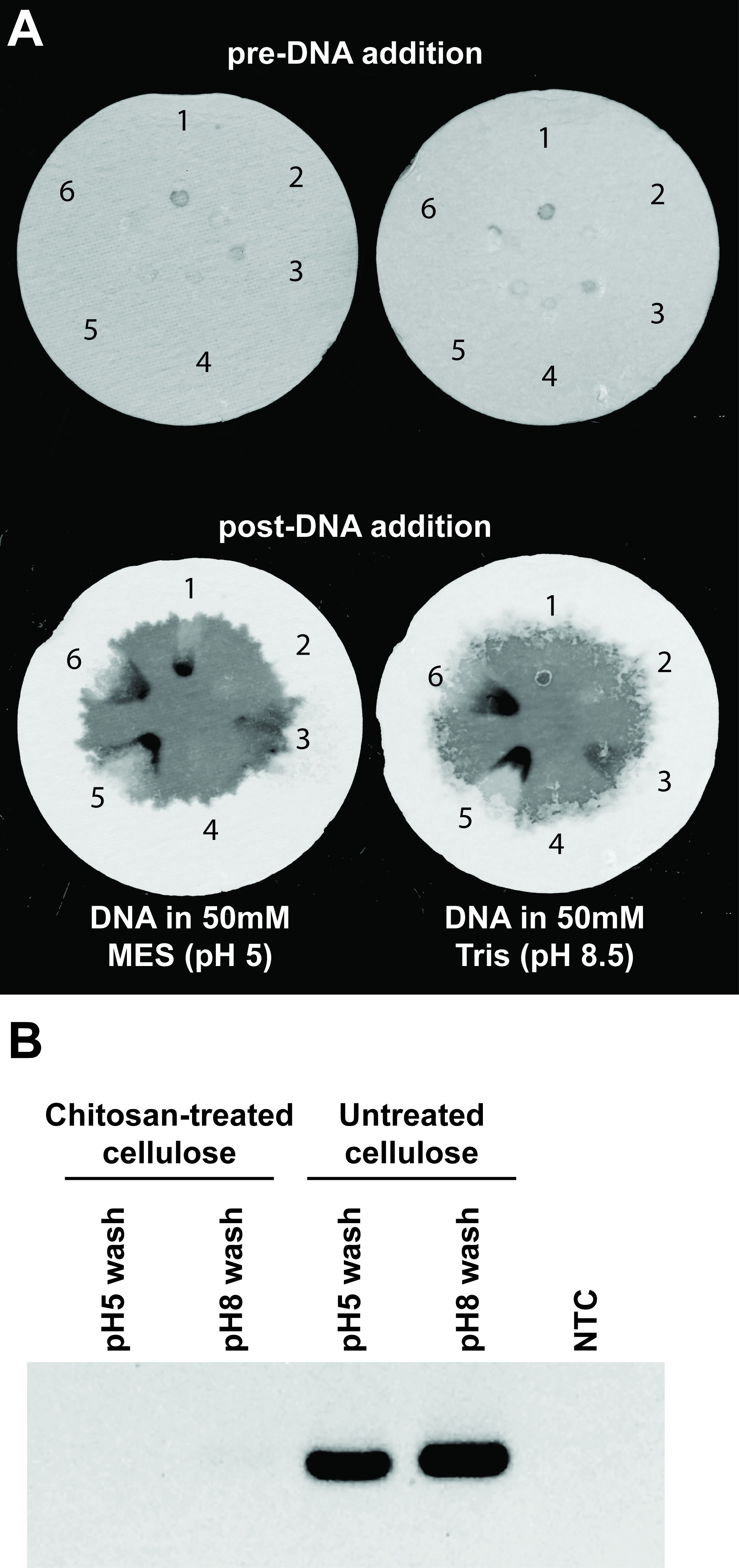

Supplement: S1 Fig — (A) GelRed-labelled salmon sperm DNA in pH5 (left image) or pH 8.5 (right image) buffer was added to the center of a Whatman No.1 filter disc on which the chemicals: 1.25% chitosan (1), 2.5% dopamine (2), 2.5% spermine (3), 2.5% polyvinylpyriliodone (4), 1.25% polyethylenimine (5), and 3-Aminopropyl-trimethoxysilane (6) had been spotted. The filters were viewed under UV light before (upper images) and after (lower images) DNA addition. (B) 3-mm diameter discs of Whatman No.1 paper that had been treated with or without 1.25% chitosan were incubated in A. thaliana genomic DNA for one minute, then washed in pH 5 or pH 8.5 buffer for one minute and then transferred to a PCR mix for amplification. 1 μl of water was used in place of the cellulose disc in the NTC. NTC, no template control. (TIF) [file pbio.2003916.s001.tif]

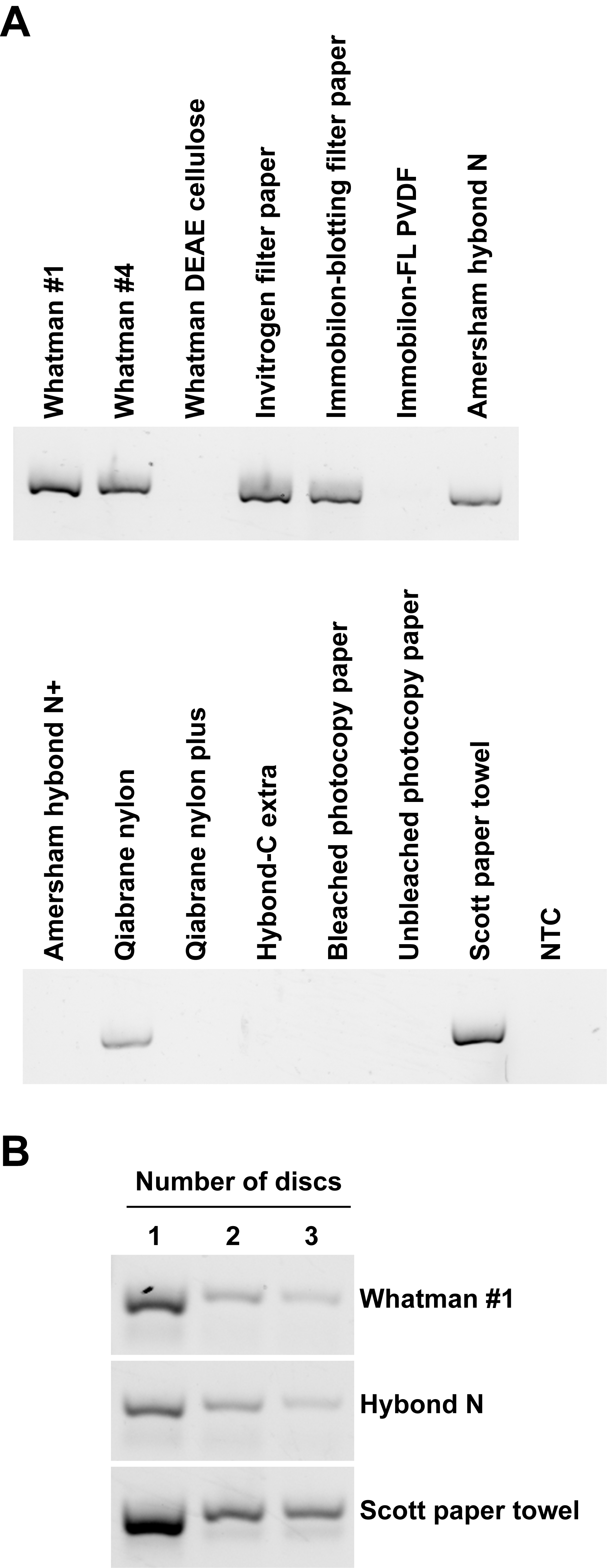

Supplement: S2 Fig — (A) Identical size fragments of a variety of sources were used to purify nucleic acids from an Arabidopsis leaf extract. The extracted nucleic acids were used for PCR amplification using primers designed for the G-protein gamma subunit 1 gene (At3g63420). (B) One, two, or three discs (3-mm diameter) of Whatman No.1, Hybond N or Scott-brand paper towel were incubated in purified Arabidopsis DNA, washed, and then used in a PCR reaction using primers designed for the G-protein gamma subunit 1 gene. (TIF) [file pbio.2003916.s002.tif]
