# Supplementary material for: Nucleic acid purification from plants, animals and microbes in under 30 seconds
Source: PLoS Biol. 2017 Nov 21;15(11):e2003916. doi: 10.1371/journal.pbio.2003916 (PMC5697807; doi:10.1371/journal.pbio.2003916)
Supplement: S1 Table — The names, sequences, target species, and source of each oligonucleotide used in this study. (DOCX) [file pbio.2003916.s005.docx]

| **Sequence (5’-3’)** | **Species** | **Target gene** | **Primer source** |
| --- | --- | --- | --- |
| GAGAGAGAGACTTCGACGACA | *Arabidopsis thaliana* | G-protein gamma subunit 1 | This study |
| GCTATCCACGAGAGACCTACG |  |  |  |
| TTGTTTGGAGCTTGCTGATG | Rice | Betaine aldehyde dehydrogenase 2 | [1] |
| CATAGGAGCAGCTGAAATATATACC |  |  |  |
| YGACTCTCGGCAACGGATA | Tomato, sugarcane, sorghum, soybean | ITS regions | [2] |
| RGTTTCTTTTCCTCCGCTTA |  |  |  |
| YGACTCTCGGCAACGGATA | tobacco, barely, wheat, mandarin, lime, lemon, passion fruit | 5.8S ribosomal RNA | [2] |
| GCGTTCAAAGAYTCGATGRTTC |  |  |  |
| AAACTCTGGTGGAGGTCCGT | Human cell line | 28S ribosomal RNA | [3] |
| CTTACCAAAAGTGGCCCACTA |  |  |  |
| ATAGGTGATTTTGGTCTAGCTACTGT | Human blood | Braf | This study |
| AGTAACTCAGCAGCATCTCAGG |  |  |  |
| AAAGCCGCATATCCCCCA | *Pseudomonas syringae* | NA | This study |
| TCAGATACCGTCTCCTCACAC |  |  |  |
| AAGGTTGATATGTCCGCACC | *Actinobacillus pleuropneumoniae* | Outer membrane lipoprotein A-like | [4] |
| CACCGATTACGCCTTGCCA |  |  |  |
| AGTTAATCCTTTGCCGAAATTTGATTCTAC | Cucumber mosaic virus | Coat protein | [5] |
| GTGCTCGATGTCAACATGAAGTACTAGCTC |  |  |  |
| TCTTATCCCATCCCCAGCAT  CAACTCCTGTACGGATTGCG | *Fusarium oxysporum* f.sp conglutinans (PCR amplification) | NA | This study |
| GGATACATGAGTGTCCCTCAAGTG  ACAACAGCAAAACACCGCTT  CTTGTCGCCTAGATCAGCTAAGTATCGAACAGTTTCTACCGATGCTGAAGG  AGCAGTGCGTCACATTACATAACCTGTCTCCATGGGACAATCATACG | Cucumber mosaic virus (LAMP amplification) | Movement protein | This study |

**References:**

1. Bradbury LMT, Henry RJ, Jin QS, Reinke RF, Waters DLE. A perfect marker for fragrance genotyping in rice. Mol Breed. 2005;16: 279-283.

2. Cheng T, Xu C, Lei L, Li CH, Zhang Y, Zhou SL. Barcoding the kingdom Plantae: new PCR primers for ITS regions of plants with improved universality and specificity. Mol Ecol Resour. 2016;16: 138-149.

3. Naito E, Dewa K, Ymanouchi H, Kominami R. Ribosomal Ribonucleic-Acid (Ribosomal-RNA) Gene Typing for Species Identification. J Forensic Sci. 1992;37: 396-403.

4. Gram T, Ahrens P. Improved diagnostic PCR assay for Actinobacillus pleuropneumoniae based on the nucleotide sequence of an outer membrane lipoprotein. J Clin Microbiol. 1998;36: 443-448.

5. Wee E, Lau H, Botella J, Trau M. Re-purposing bridging flocculation for on-site, rapid, qualitative DNA detection in resource-poor settings. Chem comm. 2015;51: 5828-5831.
